# Kinematics of turning during walking over ground and on a rotating treadmill

**DOI:** 10.1186/1743-0003-11-127

**Published:** 2014-08-23

**Authors:** Janez Pavčič, Zlatko Matjačić, Andrej Olenšek

**Affiliations:** University Rehabilitation Institute, Republic of Slovenia, Ljubljana, Slovenia

**Keywords:** Rotating treadmill, Treadmill walking, Over ground walking, Torso angle, Pelvis angle, Stride length, Intra-class correlation coefficient

## Abstract

**Background:**

After neurological injury, gait rehabilitation typically focuses on task oriented training with many repetitions of a particular movement. Modern rehabilitation devices, including treadmills, augment gait rehabilitation. However, they typically provide gait training only in the forward direction of walking, hence the mechanisms associated with changing direction during turning are not practiced. A regular treadmill extended with the addition of rotation around the vertical axis is a simple device that may enable the practice of turning during walking. The objective of this study was to investigate to what extent pelvis and torso rotations in the transversal plane, as well as stride lengths while walking on the proposed rotating treadmill, resemble those in over ground turning.

**Methods:**

Ten neurologically and orthopedically intact subjects participated in the study. We recorded pelvis and torso rotations in the transversal plane and the stride lengths during over ground turning and while walking on a rotating treadmill in four experimental conditions of turning. The similarity between pelvis and torso rotations in over ground turning and pair-matching walking on the rotating treadmill was assessed using intra-class correlation coefficient (ICC - two-way mixed single measure model). Finally, left and right stride lengths in over ground turning as well as while walking on the rotating treadmill were compared using a paired t-test for each experimental condition.

**Results:**

An agreement analysis showed average ICC ranging between 0.9405 and 0.9806 for pelvis and torso rotation trajectories respectively, across all experimental conditions and directions of turning. The results of the paired t-tests comparing left and right stride lengths showed that the stride of the outer leg was longer than the stride of the inner leg during over ground turning as well as when walking on the rotating treadmill. In all experimental conditions these differences were statistically significant.

**Conclusions:**

In this study we found that pelvis rotation and torso rotation are similar when turning over ground as compared to walking on a rotating treadmill. Additionally, in both modes of turning, we found that the stride length of the outer leg is significantly longer than the stride length of the inner leg.

**Electronic supplementary material:**

The online version of this article (doi:10.1186/1743-0003-11-127) contains supplementary material, which is available to authorized users.

## Background

Walking is an elementary way to move in predominantly static domestic and dynamic public environments. While this may be trivial to healthy people it could be a substantial challenge to people with brain or spinal cord injuries or diseases. Spasticity, muscle weakness, diminished dynamic stability, osteoporosis and associated physical inactivity not only make walking cost-ineffective, but also seriously hinder locomotion mechanisms, and even increase the risk of falling[1]. Gait rehabilitation in neurologically impaired individuals takes advantage of neuroplasticity, a capability of the brain to form new neuron pathways and synapses[2–5]. However, since the human brain is the most susceptible to such changes early after neurological injury and deteriorates thereafter, it is imperative to commence gait rehabilitation as soon as possible in order to maximize the chance for rehabilitation success.

The conventional task-oriented approach to gait rehabilitation assumes that the physiotherapist manually guides a patient to properly complete many repetitions of a particular movement. In the early stages of gait rehabilitation, the training primarily focuses on regaining muscle strength and the restoration of cyclical leg movement. Eventually gait training proceeds with more demanding weight bearing exercises (e.g. standing up and sitting down, step climbing), combined with increasingly more challenging gait maneuvers (e.g. gait initiation and stopping, walking straight and turning) which then further improves gait function. Such a rehabilitation program continually puts to the test the neurological control of the patient and requires a substantial amount of focus and effort on the part of the therapists as well. While providing corrective actions to the limbs, the therapist must at the same time ensure fall safe training conditions by providing manual support to the pelvis and trunk when necessary. Fortunately, nowadays therapists can choose from a large assortment of assistive and rehabilitation devices that unburden them from having to manually support the patients[6–12]. One of the most accepted and a widespread training device is a treadmill. Small space requirements, reliable speed control and integrated body weight support that facilitates training of neurologically impaired individuals, seem to successfully counterbalance potential disadvantages of treadmill walking as compared to over ground walking. Namely, there is an ongoing debate in the literature as to whether treadmill training adequately emulates over ground walking. Evidence exist that treadmill walking induces somewhat different gait kinematics as opposed to natural walking on the ground[6, 13]. On the other hand, other studies have concluded that over ground and treadmill walking are basically equal regarding kinematic and kinetic data, as long as treadmill speed is constant and subjects are familiarized with the treadmill[14, 15]. Unfamiliarity could be the reason for quicker cadence, shorter stride lengths and differences in some joint kinematics during treadmill walking[16–18]. One distinctive characteristic of a treadmill is that it only supports training of gait mechanisms that are associated with straight walking. However, according to some studies turning can represent from between 8% to 50% of all walking activities, depending on the environment[19]. Furthermore, in turning people recruit muscle groups that are less involved in straight walking. Hence turning requires specific control strategies that need to be practiced. In the elderly, where walking ability is progressively getting weaker due to decreased acuity of the vestibular and somatosensory system as well as decreased flexibility and muscle tone due to loss of motor neurons, it is assumed that falling while turning is 7.9 times more likely to result in hip fracture, than falling while walking straight ahead[20]. The elderly successfully mitigate the associated risks by turning slowly and taking additional steps[21]. Observations of turning in healthy control subjects further indicate that turning at a self-selected speed is significantly slower, features shorter stride lengths[22, 23], and exhibits a larger amount of knee flexion during stance phase on the inner leg, and a larger plantar flexion on the outer leg[22], as compared to walking in a straight line. Consequently, there is a strong need for a rehabilitation tool that would allow training of turning maneuvers, as well as straight walking in fall-safe training conditions, that should however be constrained to a limited space in order to be feasible for use in a clinical environment. Until appropriate technical solutions are developed, such training remains exclusively in the domain of over ground gait training under supervision of skillful therapists.

One possible approach to enable the practice of turning would be to extend a regular treadmill with an additional degree of freedom that would allow the treadmill to rotate around the vertical axis of the center of the treadmill, thus forcing a subject to change the direction of walking in accordance to the angular displacement of the treadmill. Such a rotating treadmill may have the potential to create a fall-safe training environment, which would allow neurologically impaired individuals to practice similar control mechanisms that are typically present in over ground turning during walking.

The objective of this exploratory study was to investigate how well and to what extent walking on the proposed rotating treadmill resembles over ground turning. In particular we were primarily interested in i) how well pelvis and torso rotations in transversal plane in over ground turning (OG) relate to pelvis and torso rotations in transversal plane while walking on rotating treadmill (RT); and ii) the relation between left and right stride lengths during OG on one hand, and the relation between left and right stride lengths while walking on a RT on the other. Several studies have shown that when turning at moderate speeds during over ground walking, differences exist i) in the rotation of the pelvis and torso in the transversal plane, and ii) in the stride lengths of both legs, where the stride of the outer leg is typically longer than the stride of the inner leg[22–26].

## Methods

### Rotating treadmill

**Figure 1:**
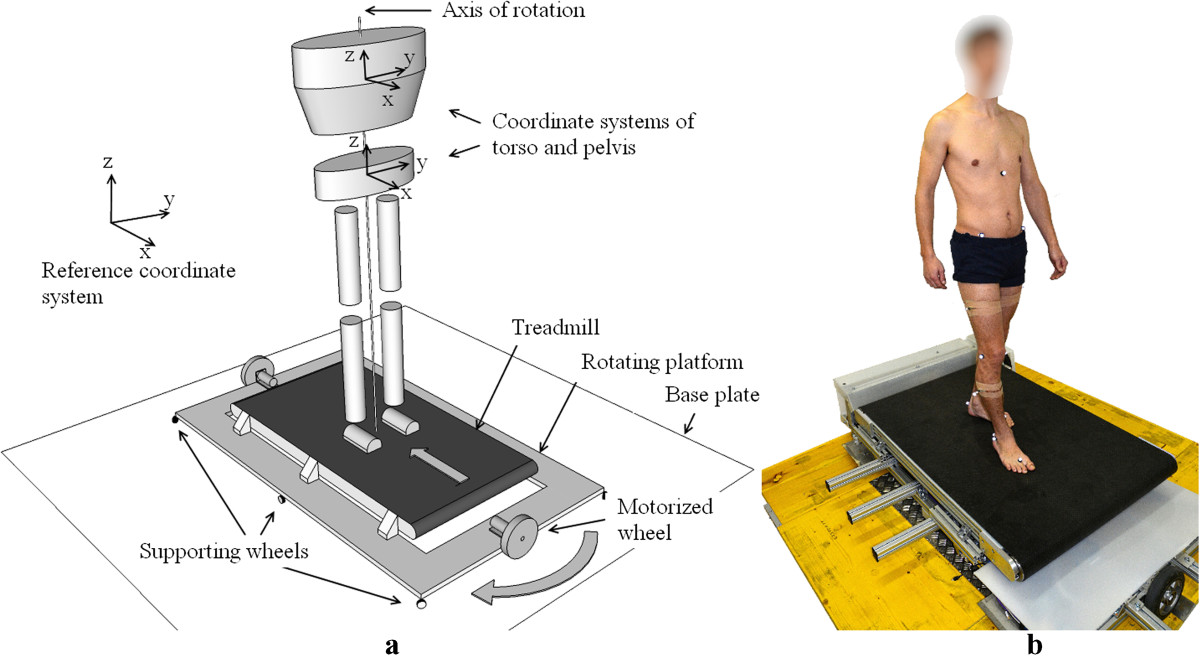
**Rotating treadmill; a) conceptual setup and b) actual design.**

The rotating treadmill is composed of three main components: base plate, rotating platform and conventional treadmill. The base plate is made of ply shuttering panels with a combined surface area of 2 × 2 m^2^. It carries a bearing unit with the axis of rotation aligned vertically, and a system of brushes mounted on top of the bearing unit to provide access to the signal and power lines of the rotating treadmill. The rotating side of the bearing unit is further attached to the rotating platform, this confines the feasible range of movement of rotating platform to only one rotation about the vertical axis. To minimize the load on the bearing unit, six smaller and two larger wheels support the rotating platform during turning. All the supporting wheels have their axes of rotation aligned with the circumference of the circle that the rotating platform outlines while turning. One of the larger wheels is equipped with a DC motor, an encoder and a corresponding control unit (all Maxon Motor ag) that relays angular propulsion to the rotating platform. The direction and speed of rotation is set electronically through a change in voltage polarity, and amplitude ranging from -10 V to +10 V, respectively. Finally, the rotating platform carries a commercially available treadmill (Energetics) with a walking area of 1.3 × 0.45 m^2^ with the center of walking aligned with the vertical axis of the bearing unit. The conceptual setup and the actual design of the rotating treadmill is shown in Figure 1.

### Participants and data acquisition

We invited ten healthy male subjects aged from 24 – 44 years (mean ± SD: 31.5 ± 6.04 years) and with no known neurological or orthopedic disorders to participate in the study. 17 reflective markers were placed on the selected anatomical landmarks of each subject by a skillful physiotherapist according to the Conventional Gait Model[27, 28] (10th thoracic vertebrae, sternum, left and right anterior superior iliac spine, sacrum, left and right mid-thigh, left and right knee joint, left and right mid-shank, left and right lateral malleoli, left and right second metatarsal head, left and right heel). The Vicon Motion System (Oxford Metrics Ltd) was used to record the gait kinematics and the spatial parameters at a sample rate of 100 Hz. To ensure a fixed position of the markers during kinematic data collection, all subjects wore low-rise shorts that allowed markers to be attached directly to each subjects’ skin. All subjects were informed about the experiment protocol and signed a consent form. The study was approved by the Slovenian national ethics committee.

### Protocol

For the experimental protocol, we assumed four separate experimental conditions of turning, each utilizing the same speed of walking (linear velocity), and the same speed of turning (angular velocity) for both modes of walking (OG and RT). We first arbitrarily selected two linear velocities *v*_*1*_ = 0.6 m/s and *v*_*2*_ = 1 m/s, reflecting a slow and a moderate speed of walking, and then combined them with two angular velocities *ω*_*1*_ = 30°/s and *ω*_*2*_ = 40°/s. This implied that for completing one full rotation of 360° one would require *t*_*1*_ = 12 s and *t*_*2*_ = 9 s respectively. While this is subject to angular velocity control in RT mode, in OG mode, the selected linear velocities (*v*_*1*_ and *v*_*2*_) and times required for one full rotation (*t*_*1*_ and *t*_*2*_), correspond to four circles with a corresponding circle radii *r*_*1*_ = 1.15 m, *r*_*2*_ = 1.91 m, *r*_*3*_ = 0.86 m and *r*_*4*_ = 1.43 m respectively, which subjects had to complete in a given time. To assure all conditions in OG mode were met, all circles were marked on the floor within the recording volume of the Vicon motion capture system – the subjects were obligated each time to step on the line with the inner leg – and the time needed to complete one full circle was monitored. Details of the experimental conditions are gathered in Table 1. To illustrate, according to Table 1 in experimental condition ω_1_v_1_, we evaluated similarities between OG and RT turning by comparing i) RT mode of walking with subjects walking on RT with linear velocity set to *v*_*1*_ = 0.6 m/s and angular velocity set to *ω*_*1*_ = 30°/s, ii) to pair-matching OG turning with subjects walking over ground in circles with *r*_*1*_ = 1.15 m, completing one circle in *t*_*1*_ = 12 s.

**Table 1:** **Experimental conditions**

	Turning on RT		OG turning		
Experimental condition	Angular velocity of RT	Linear velocity (speed of walking)	Radius of a circle	Circle time	Linear velocity (speed of walking)
ω_1_v_1_	30°/s	0.6 m/s	1.15 m	12 s	0.6 m/s
ω_1_v_2_	30°/s	1.0 m/s	1.91 m	12 s	1.0 m/s
ω_2_v_1_	40°/s	0.6 m/s	0.86 m	9 s	0.6 m/s
ω_2_v_2_	40°/s	1.0 m/s	1.43 m	9 s	1.0 m/s

Turning maneuvers in experimental conditions (ω_1_v_1_, ω_1_v_2_, ω_2_v_1_, ω_2_v_2_) were first executed in a counter clockwise (CCW), followed by a clockwise (CW) direction of turning. Each subject commenced the session by walking OG in circles according to the experimental conditions in Table 1. Based on the time needed to complete one full circle, verbal instructions were given to the subject until the subject settled at desired circle time. Measurement of the gait kinematics started only after circle time remained within ten percent of the desired value for at least two circles (thus achieving the desired gait velocity). In each experimental condition, subjects had to complete at least three full circles or continue walking until a minimum of ten strides were obtained for the left and right leg. If necessary, multiple trials were recorded to meet this requirement. We concluded the session with walking on the rotating treadmill (RT) with linear and angular velocities set according to Table 1. Again, subjects had to complete at least three full circles in each experimental condition or continue walking until a minimum of ten strides were obtained for left and right leg. In our analysis we considered only strides when gait velocity did not deviate more than ten percent from desired gait velocity –cases involving larger deviations in the strides were not considered valid, and were excluded from further analysis.

Throughout the experiment we assumed that subjects had no prior experience with treadmill walking, particularly in relation to walking on a rotating treadmill. For this reason all subjects were given five minutes to get acquainted with the new experimental conditions. Data collection commenced only after subjects confirmed that they were comfortable with walking on the rotating treadmill.

### Data analysis

#### Kinematics – pelvis and torso rotations in the transversal plane

Marker trajectories were filtered using Vicon’s built-in Woltring quintic spline algorithm. We detected gait events (foot strike and foot off) in an offline analysis, by inspecting the three dimensional trajectories of markers designating left and right heel (LHEE and RHEE respectively) as well as left and right second metatarsal head (LTOE and RTOE respectively) frame by frame in the Vicon Nexus environment. Given that all subjects were healthy adults, they all initiated the stance phase by contacting the heel first, followed by forefoot contact, and finally by lifting the heel off the ground first and toes last. Therefore, in OG mode we designated foot strike to be the time instance when LHEE or RHEE came to rest, and assumed positions closest to the ground, thus completing the swing. Foot off was considered as a time instance of when LTOE or RTOE was elevated and set in motion, thus initiating the swing. On the other hand, in RT mode we designated foot strike to be the time instance when LHEE or RHEE after progressing forward in the swing reversed their directions of movement and assumed positions closest to the treadmill belt, whereas the foot off was considered as a time instance of when LTOE or RTOE reversed their direction, and elevated in order to initiate the forward swing. All gait events were exported along with marker trajectories to Matlab (The MathWorks, Inc.) for further processing. In our analysis we primarily focused on pelvis and torso rotation in the transversal plane (we will refer to them simply as torso rotation and pelvis rotation and omit related axis/plane of rotation unless necessary). According to this, coordinate frames were designated to the torso and the pelvis. By following the Conventional Gait Model[27, 28], the origin of the pelvis coordinate system was defined at the midpoint between the RASI and the LASI markers (denoting left and right anterior superior iliac spine respectively). The Y axis was defined to be the direction from the RASI marker to the LASI marker, the Z axis was perpendicular to the plane formed by the RASI, LASI and SACR markers (denoting sacrum), and the X axis completed the right-handed coordinate system of the pelvis. The coordinate system of the torso was defined in a similar way: first the X axis was defined as the direction from the T10 marker (denoting 10th thoracic vertebrae) to the STRN marker (denoting sternum), the Y axis was perpendicular to the X axis and the vector denoting the direction from the T10 marker to the SACR marker, whereas the Z axis completed the right-handed coordinate system of the torso. Illustration of the pelvis and torso coordinate systems is shown in Figure 1a. Finally Cardan angles (sequence y – tilt, x – oblique and z - rotation) were used to describe the orientations of the pelvis and torso with respect to the reference (global) coordinate system for every sample time, and was gathered separately for each gait cycle i.e. stride.

Stride is defined as a time between two consecutive heel strikes of the same foot. When turning CCW we observed strides indicated by two consecutive right heel strikes, whereas when turning CW we observed strides indicated by two consecutive left heel strikes. For each gait cycle, the values of pelvis and torso rotation were time-normalized to a percentage of the gait cycle. To acknowledge that each stride starts at an arbitrary pelvis orientation with respect to the reference coordinate system, the pelvis rotation was offset at each percent of stride against the pelvis rotation at the beginning of the stride (0% of gait cycle). Likewise, to acknowledge that each stride starts at an arbitrary torso rotation with respect to the reference coordinate system, but at the same time to take into account the relative movement of the torso with respect to the pelvis torso rotation, an offset at each percent of stride against pelvis rotation at the beginning of stride (0% of gait cycle) was applied. After processing, pelvis and torso rotations for a minimum of five gait cycles per subject for OG turning, and a minimum of nine gait cycles per subject for RT walking, were obtained per experimental condition and per direction of turning. For all strides of a particular subject that were related to a specific combination of mode of walking *mw*∈{OG,RT}, experimental condition *ec*∈{ω_1_v_1_, ω_1_v_2_, ω_2_v_1_, ω_2_v_2_}, and direction of turning *dt*∈{CW, CCW}, we then separately averaged pelvis and torso rotation patterns to obtain the subject’s specific representative pelvis rotation γ_*i,P,mw,ec,dt*_ and torso rotation pattern γ_*i,T,mw,ec,dt*_. For example, γ_*3,P,OG,ω1v1,CCW*_ indicated the averaged pelvis rotation for a particular subject during OG turning in CCW direction, with a speed of walking and angular velocity as determined by experimental condition ω_1_v_1_.

We used intra-class correlation coefficient (ICC - two-way mixed single measure model) as a measure of the similarity between pelvis rotations when turning OG (γ_*i,P,OG,ec,dt*_) and when turning on the RT (γ_*i,P,RT,ec,dt*_), and as a measure of the similarity between torso rotation patterns when turning OG (γ_*i,T,OG,ec,dt*_) and when turning on the RT (γ_*i,T,RT,ec,dt*_), giving *ICC*_*i,P,ec,dt*_ and *ICC*_*i,T,ec,dt*_ respectively[29]. Finally *ICC*_*i,P,ec,dt*_ and *ICC*_*i,T,ec,dt*_ were averaged across all subjects to show the average absolute agreement with respect to the specific experimental conditions and direction of turning, these were labeled as *ICC*_*P,ec,dt*_ and *ICC*_*T,ec,dt*_.

#### Spatial parameters - stride length

**Figure 2:**
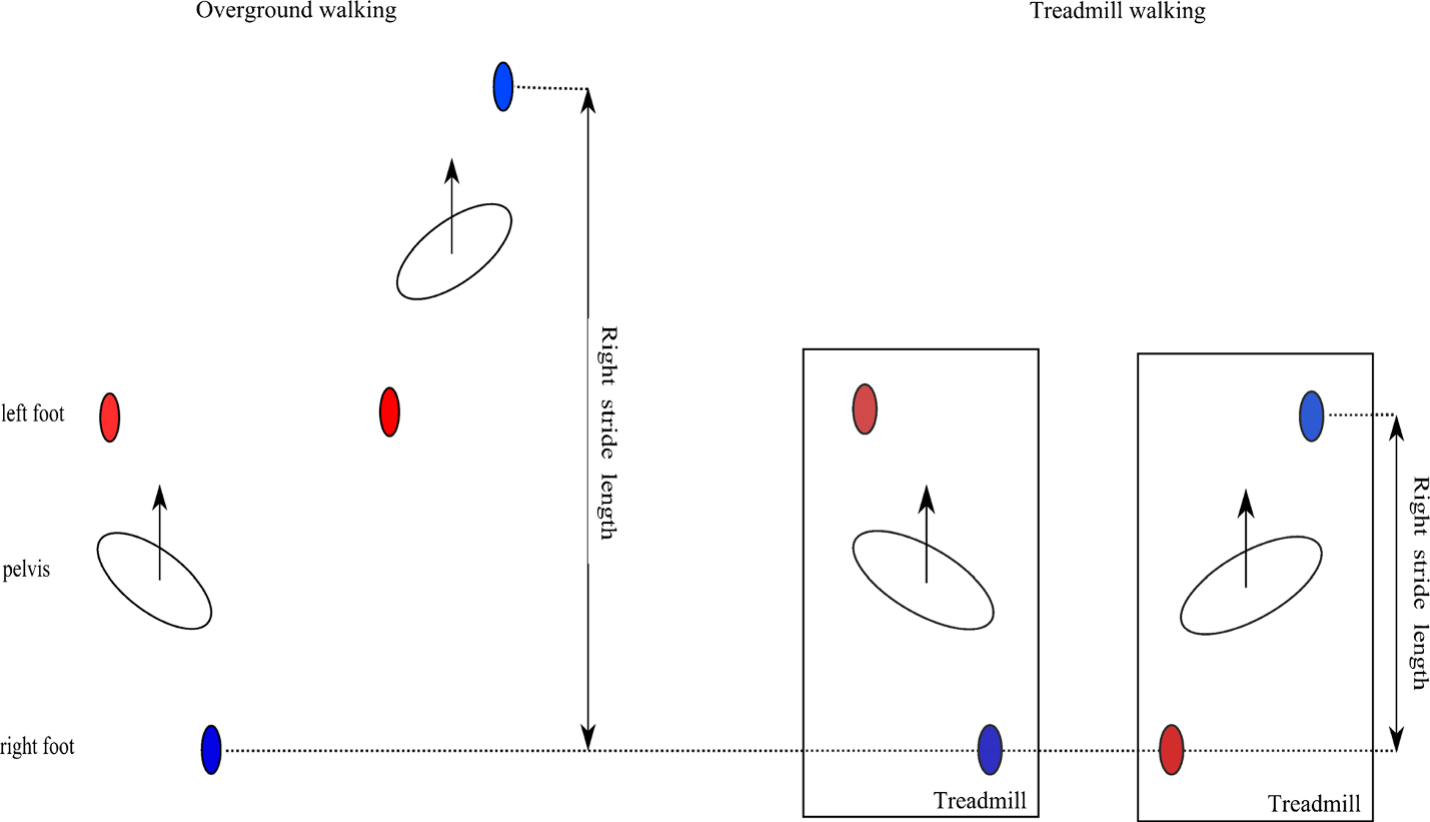
**Definition of the stride length in over ground and treadmill walking.**

Stride length is formally defined as the distance made by the same foot between two consecutive foot contacts. Such a definition is intuitive for over ground walking, but not practical in treadmill walking, because of the relative movement of the stance leg and consequently approximately the same position of contact. For this reason we defined left/right stride length as the distance traveled by the LANK/RANK marker (denoting left/right ankle joint) between foot-off of the left/right foot and the subsequent foot strike of the same foot. Depending on the mode of walking such a definition of stride length leaves us with somewhat different implications for over ground walking and walking on a treadmill as schematically shown in Figure 2. In over ground walking (Figure 2, left side), stride length reflects the distance traveled by the swing leg, while the stance leg remains stationary on the ground. Due to the fixed position of the stance leg such a definition of the stride length should give the same result as in formal definition of the stride length. On the other hand, we acknowledge that when walking on a treadmill (Figure 2, right side), stride length reflects the distance traveled by the swing leg, while the stance leg together with the treadmill belt moves in the opposite direction. Such a definition of stride length does not account for relative movement of the stance leg, and is therefore expected to be shorter compared to the stride length in OG walking. Nonetheless, since in our analysis we were investigating the difference between left and right stride lengths under the same experimental conditions (therefore also under the same mode of walking: either mw = OG or *mw* = RT), such a definition of stride length is completely sufficient – when *mw* = RT the same relative movement of the stance leg is present in the left and right stride, and has therefore no effect in our analysis.

The representative left/right stride lengths for a particular subject and for a particular mode of walking, together with the experimental conditions, as well as the direction of turning, was calculated by averaging left/right stride lengths across corresponding valid strides. According to this, left and right stride lengths were labeled as *LSL*_*i,mw,ec,dt*_ and *RSL*_*i,mw,ec,dt*_, where *i* indicates the consecutive number of particular subjects (*i∈*{1…10}), *mw* denotes mode of walking (*mw*∈{OG,RT}), *ec* indicates experimental condition (*ec*∈{ω_1_v_1_, ω_1_v_2_, ω_2_v_1_, ω_2_v_2_}), and *dt* denotes the direction of turning (*dt*∈{CW, CCW}). Left and right stride lengths were then compared with paired t-tests separately for each *mw*, *ec* and *dt* (e.g. *LSL*_*i,RT,ω1v1,CCW*_ compared to *RSL*_*i,RT,ω1v1,CCW*_)*.*

## Results

### Kinematics – pelvis and torso rotations in the transversal plane

**Figure 3:**
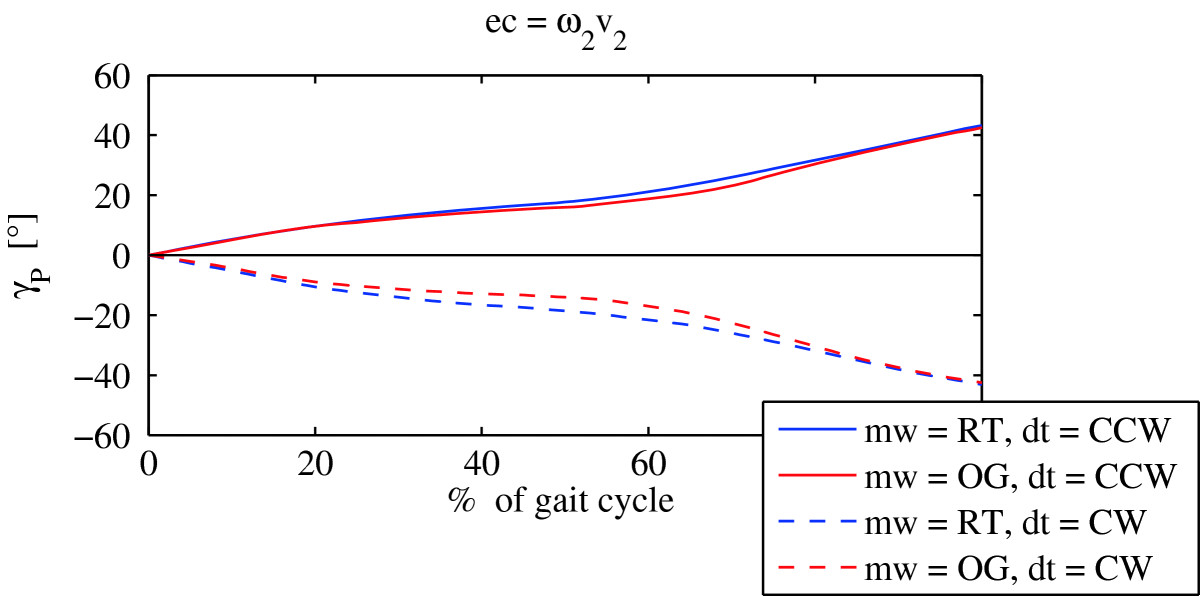
**Mean pelvis rotations for the group of healthy adults (** ***mw*** **– mode of walking;** ***ec*** **– experimental condition;** ***dt*** **– direction of turning).**

Figure 3 compares mean rotation angles of the pelvis between the *mw =* OG mode of walking and the *mw =* RT mode of walking as recorded in experimental condition ec = ω_2_v_2_. It shows that the movement of the pelvis in the transversal plane is almost identical in both modes of walking (*mw* = OG and *mw* = RT). In the first 20% of the gait cycle, the pelvis rotation in the *mw =* OG mode of walking is aligned with the pelvis rotation in the *mw =* RT mode of walking. It is only until the middle of the gait cycle when differences of no more than 5° become evident in all experimental conditions. Furthermore, in all experimental conditions, the pelvis rotation immediately after the beginning of the gait cycle displays movement of the pelvis in the direction of walking. In the middle of the gait cycle (approximately 40% - 60% of the gait cycle), pelvis rotation starts to slow down and almost stops, with respect to the world coordinate system and also with respect to the rotation of the treadmill around z-axis, and later accelerates to catch-up with the direction of turning at the end of gait cycle. Similar observations were identified also in other experimental conditions.

**Figure 4:**
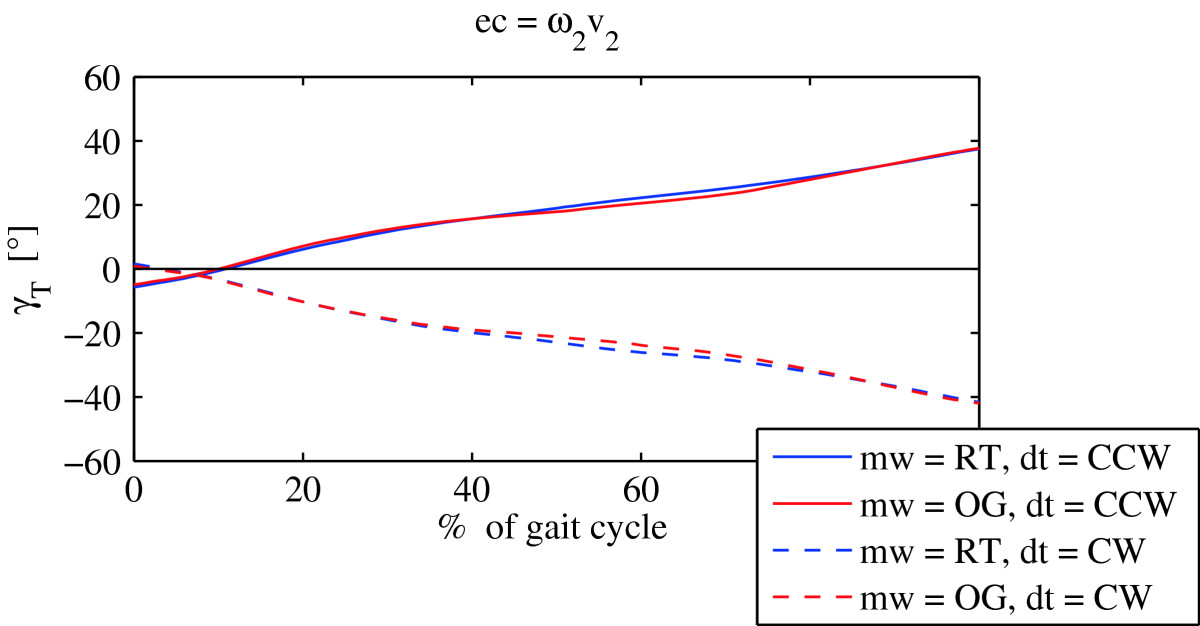
**Mean torso rotations for the group of healthy adults (** ***mw*** **- mode of walking;** ***ec*** **– experimental condition;** ***dt*** **– direction of turning).**

Similarly, Figure 4 compares torso rotations during the *mw* = OG mode of walking, and during the *mw* = RT mode of as recorded in experimental condition ec = ω_2_v_2_. It again shows that the movement of the torso in the transversal plane is aligned in both modes of walking (*mw* = OG and *mw* = RT), and that the differences between both modes of walking do not exceed 3°. Furthermore the torso rotation pattern shows considerable similarity to the pelvis rotation pattern. At the beginning of the gait cycle, the torso lags behind the pelvis, it does however move into the direction of turning. Similarly to the pelvis rotation, at approximately 30% of gait cycle, the torso rotation starts to slow down, and accelerates after 60% of gait cycle in order to catch-up with the direction of turning at the end of the gait cycle. Similar observations were identified also in other experimental conditions.

The results of the agreement analysis are presented in Table 2. They show similar average ICC values in all experimental conditions and directions of turning. Across the segment of interest (pelvis or torso), experimental conditions (ω_1_v_1_, ω_1_v_2_, ω_2_v_1_, ω_2_v_2_), and directions of turning (CCW and CW), the average ICC ranged between 0.9405 and 0.9806 with standard deviation not exceeding 0.07.

**Table 2:** **Results of agreement analysis**

Experimental condition	*ICC*_*P,ec,dt*_		*ICC*_*T,ec,dt*_	
	*dt*		*dt*	
	CCW	CW	CCW	CW
ω_1_v_1_	0.9409 (0.0512)	0.9477 (0.0674)	0.9428 (0.0587)	0.9609 (0.0378)
ω_1_v_2_	0.9573 (0.0288)	0.9405 (0.0435)	0.9797 (0.0198)	0.9622 (0.0199)
ω_2_v_1_	0.9510 (0.0639)	0.9755 (0.0243)	0.9534 (0.0623)	0.9745 (0.0264)
ω_2_v_2_	0.9704 (0.0243)	0.9591 (0.0393)	0.9806 (0.0170)	0.9763 (0.0176)

Group average intra-class correlation coefficients and standard deviations.

### Spatial parameters - stride lengths

**Figure 5:**
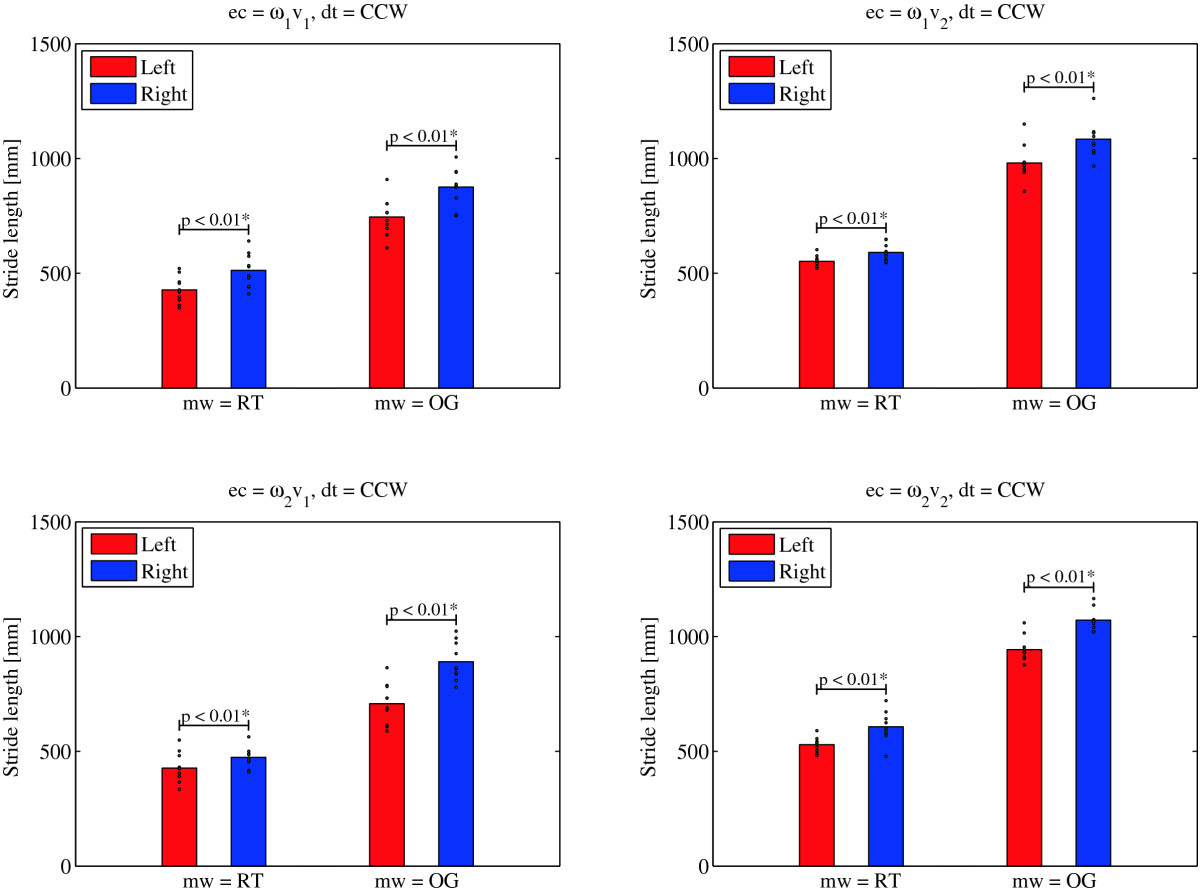
**T-test: left and right stride length for the group of subjects –** ***LSL***_***mw,ec,CCW***_ **and** ***RSL***_***mw,ec,CCW***_ **(denoted by vertical bars) and** ***LSL***_***i,mw,ec,CCW***_ **and** ***RSL***_***i,wm,ec,CCW***_ **and for individual subjects (denoted by dots) in** ***mw*** **= RT and** ***mw*** **= OG,** ***ec = ω***_***1***_***v***_***1***_***, ec = ω***_***1***_***v***_***2***_***, ec = ω***_***2***_***v***_***1***_ **and** ***ec = ω***_***2***_***v***_***2***_ **in** ***dt =*** **CCW direction of turning.**

**Figure 6:**
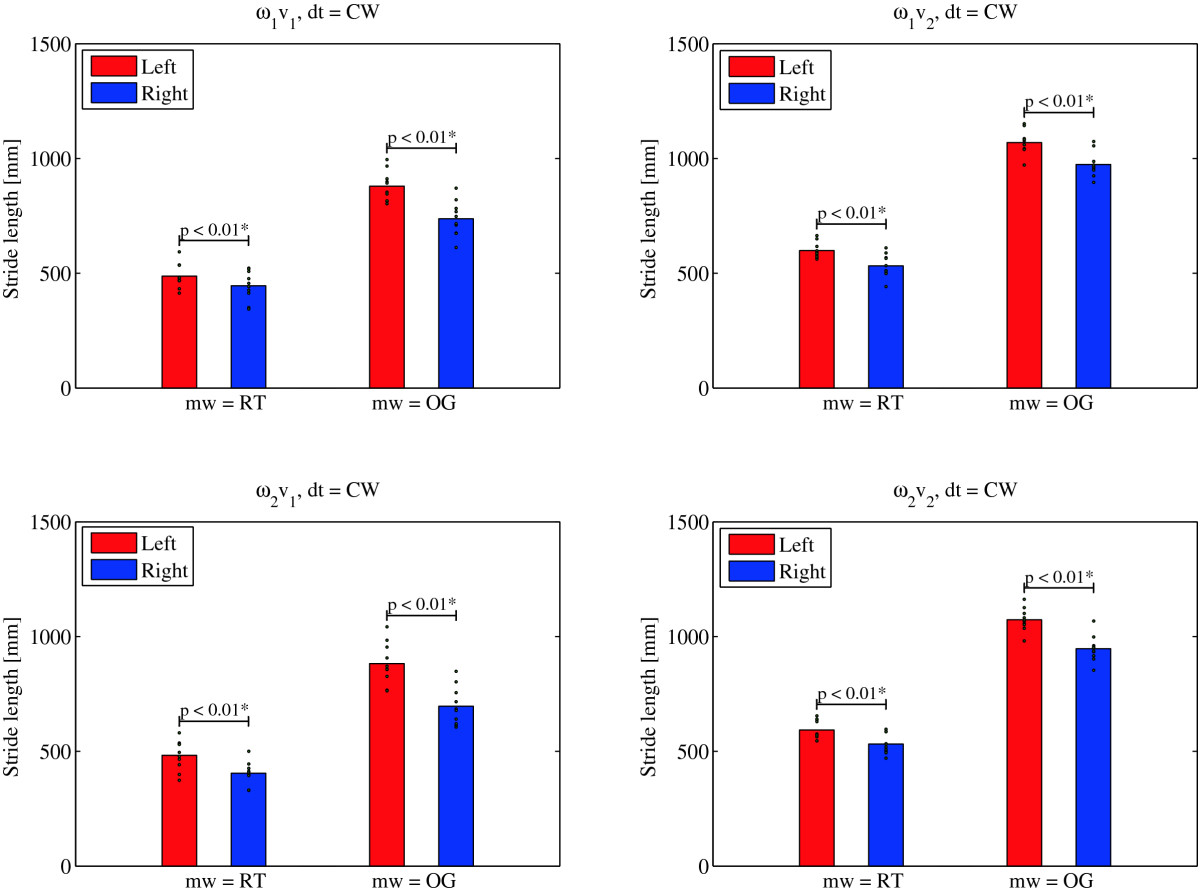
**T-test: left and right stride length for the group of subjects –** ***LSL***_***mw,ec,CCW***_**and** ***RSL***_***mw,ec,CCW***_**(denoted by vertical bars) and** ***LSL***_***i,mw,ec,CCW***_**and** ***RSL***_***i,wm,ec,CCW***_**and for individual subjects (denoted by dots) in** ***mw*** **= RT and** ***mw*** **= OG,** ***ec = ω***_***1***_***v***_***1***_***, ec = ω***_***1***_***v***_***2***_***, ec = ω***_***2***_***v***_***1***_**and** ***ec = ω***_***2***_***v***_***2***_**in** ***dt =*** **CW direction of turning.**

Figure 5 shows the paired t-test results when comparing left and right stride lengths during the CCW direction of turning. Right strides are in all experimental conditions longer than left strides regardless of whether *mw* = OG or *mw* = RT. These differences are statistically significant (p < 0.01). We found the opposite to be valid in the CW direction of turning. From Figure 6 we can conclude that in all experimental conditions associated with CW turning, left strides are longer regardless of whether *mw* = OG or *mw* = RT. The differences are again statistically significant (p < 0.01).

**Figure 7:**
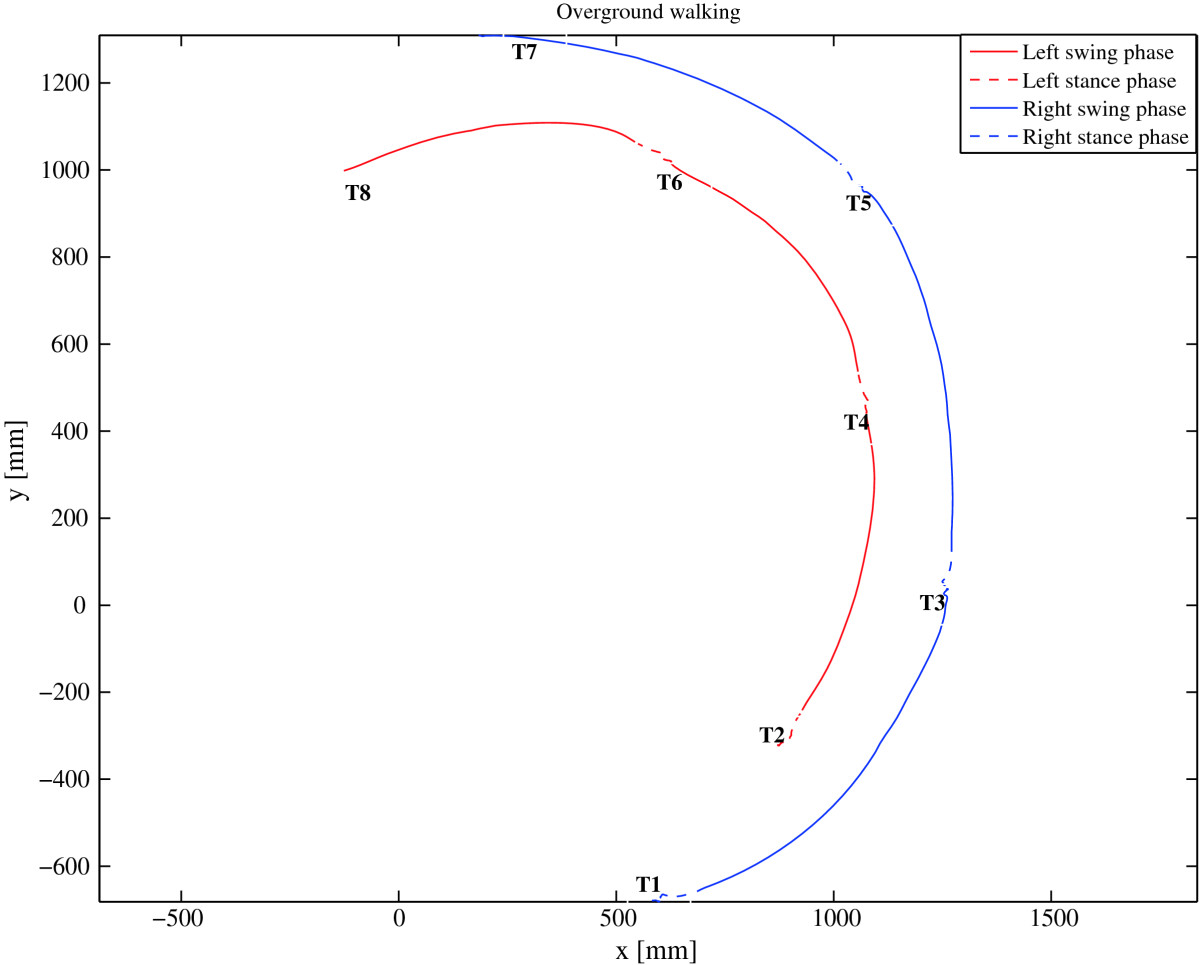
**Left (inner) and right (outer) foot position in the transversal plane for the representative trial in over ground turning in the CCW direction with respect to the relevant gait events (T1…T8) indicating beginning/end of stance and swing phases.**

**Figure 8:**
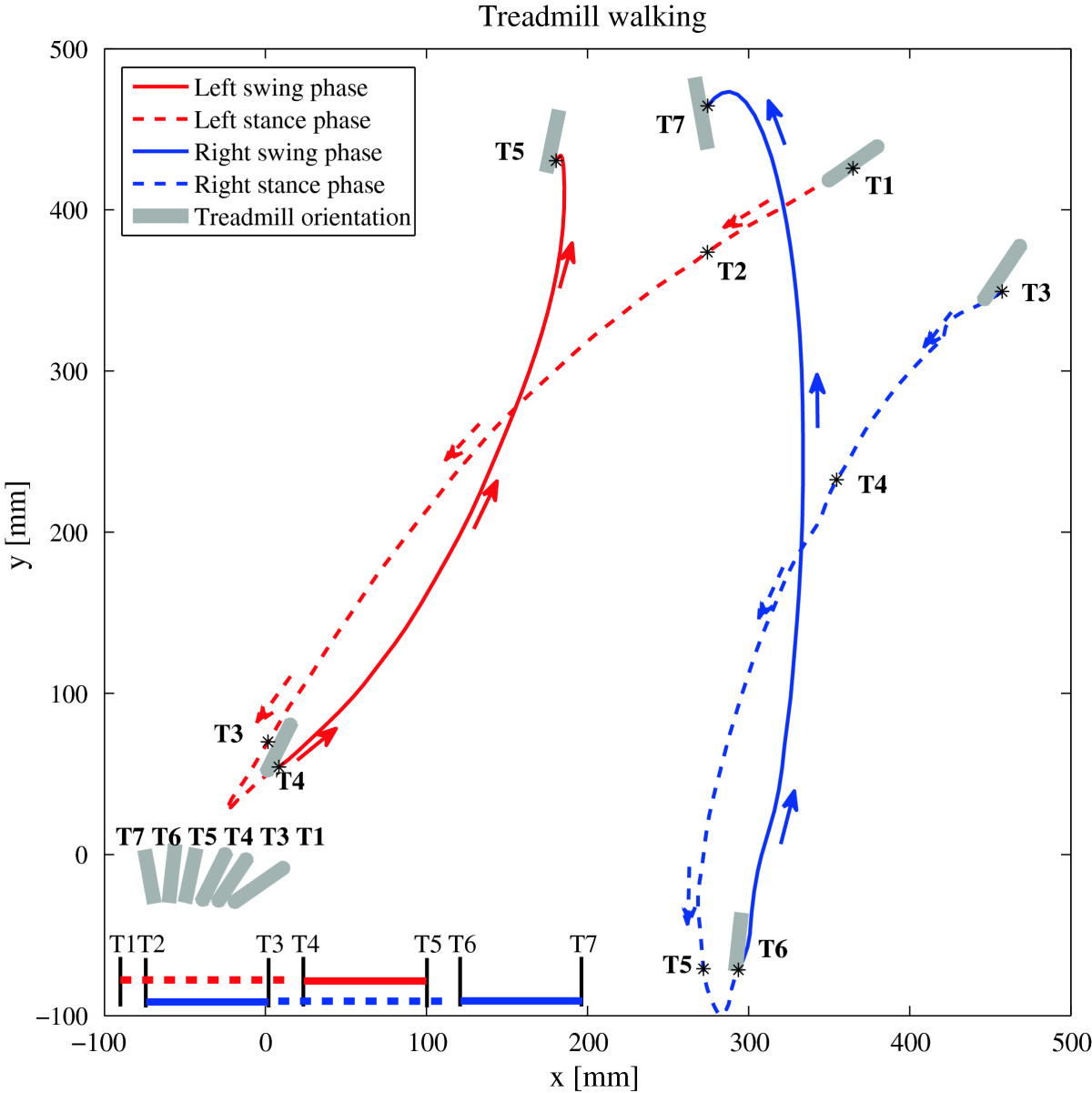
**Left (inner) and right (outer) foot positions in the transversal plane during the duration of a representative gait cycle while turning on the rotating treadmill in the CCW direction and with respect to the relevant gait events: T1 – left foot contact, T2 – right foot off, T3 – right foot contact, T4 – left foot off, T5 – left foot contact, T6 – right foot off, T7 – right foot contact.** * relates the exact timing of selected gait events with corresponding positions of left and right foot.

Figures 7 and8 further support the findings of the statistical analysis. They show the movement of the left and right foot for the representative trial and gait cycle respectively as defined by LANK and RANK (denoting left and right ankle joint) respectively in the transversal plane while turning over ground (Figure 7), and on the rotating treadmill (Figure 8) in the CCW direction. The comparison of both paths shows that in over ground turning (Figure 7), the right (outer) foot outlines a circle with a larger radius than the left (inner) foot, and that the path traveled by the right foot in the swing phase (solid section of the path) is considerably longer. Turning on the rotating treadmill in the CCW direction (Figure 8) on the other hand, displays i) movement of the stance leg with respect to the global coordinate system in the stance phase (dashed section of the path), and ii) movement of the swing leg with respect to the global coordinate system in the swing phase. Trajectories describing the paths of the left and right stance foot show that the left (inner) foot outlines a longer path in the stance phase than the right (outer) leg does in the stance phase. However, from the positions of the feet in the swing phase, we notice that the distance traveled by the right (outer) foot in the swing phase is considerably greater than the distance traveled by the left (inner) foot.

## Discussion

In this study we explored whether a conventional treadmill equipped with an additional degree of freedom that allows the treadmill to rotate around the vertical axis in the center of treadmill (rotating treadmill), may be considered as a suitable alternative to over ground training of turning. To confirm this, one would need to show that rotating treadmill establishes similar training conditions as typically present in over ground training of turning, thus demanding from the subject that they utilize similar walking control mechanisms, as one typically uses in over ground turning. Our study focused on the two most apparent kinematic features associated with turning at moderate speeds: i) during turning the pelvis and torso orientation should follow the direction of the turn; ii) the stride of the outer leg should be longer than the stride of inner leg.

### Kinematics - pelvis and torso rotation in the transversal plane

The relation between the torso and pelvis orientation has been extensively investigated in studies concerned with turning during gait, although most of them have focused on time and angle limited turns[24–26]. They report of the transient phenomena in the torso and pelvis rotation. Namely, at the initiation of the turning motion there is a change in the heading direction during the first two steps which reaches a steady state after the third step[30]. Others have established that there is a top-down sequence in the initiation of the segments of orientation during turning, starting at the head and continuing through the torso to the pelvis[31], and that the sequence is independent of the walking speed and the magnitude of the turn[32]. This is consistent with the pelvis and torso rotation patterns in the transversal plane as recorded in our study: in all experimental conditions associated with turning, the pelvis almost completely stops with respect to the ground in the middle of gait cycle, and starts to lag behind the torso, that on the other hand more closely follows the direction of turning.

More detailed visual inspection of the pelvis and torso rotations in the transversal plane show almost identical patterns in over ground turning, and their counterparts on the rotating treadmill. To evaluate to what degree pelvis rotation during over ground turning resemble the pelvis rotation, while walking on the rotating treadmill, and likewise to what degree torso rotation during over ground turning resemble the torso rotation, while walking on rotating treadmill, we calculated the mean ICCs for all experimental conditions and direction of turning. Since they all hold values greater than 0.94, irrespective of the experimental condition or the direction of turning (Table 2), we concluded that both modes of walking evoke similar pelvis and torso rotation patterns. This suggests that walking on the rotating treadmill can to some extent invoke similar walking and control mechanisms associated with the pelvis, that are typically present in over ground turning.

### Spatial parameters - stride length

**Figure 9:**
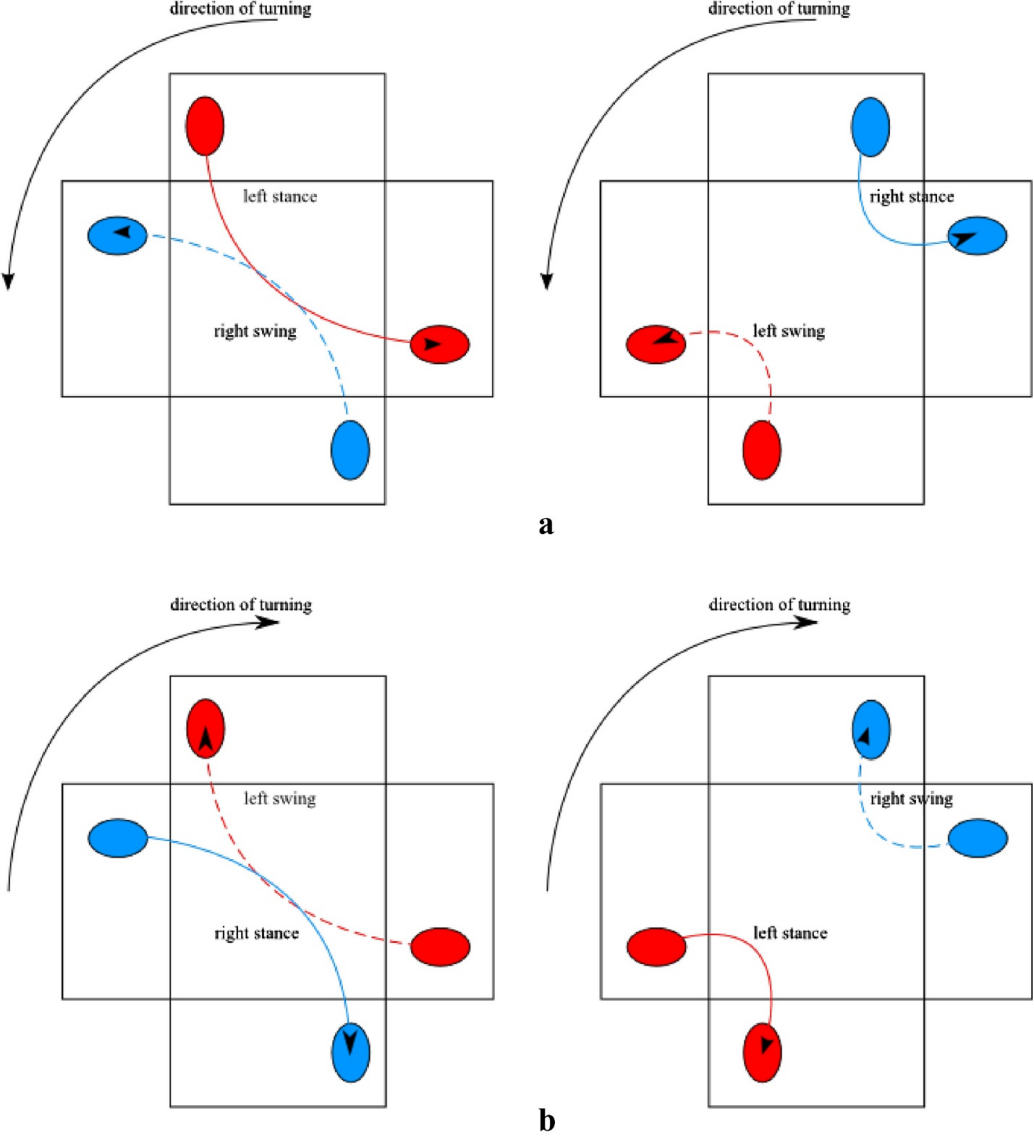
**Schematic illustration of the path outlined by the left and right foot in the stance and swing phase on the rotating treadmill in the CCW (a) and the CW (b) direction of turning.**

Studies that compared over ground turning to straight walking, reported that the outer leg outlines the circle with a larger radius than that of the inner leg[30]. It is therefore inevitable that the stride of the outer leg is longer compared to the stride of the inner leg. Our analysis of stride length is in line with these findings: when comparing stride lengths between the left and right legs as defined in Section Spatial parameters - stride length, we found that the stride length of the outer leg was significantly longer than the stride length of the inner leg, for over ground turning as well as for turning on the rotating treadmill. As illustrated in Figure 7, in over ground turning this can indeed be attributed to the shorter path of the inner (left) foot as compared to the outer (right) foot (as determined by RANK and LANK markers), due to differences in the radii of the respective circular paths. Likewise, Figure 8 outlines the path of the right and left foot for the representative gait cycle as determined by the RANK and LANK markers while walking on the rotating treadmill. Visual inspection shows that in CCW direction of turning, the path made by the outer (right) foot when in the swing phase is longer than the path made by the inner (left) foot when in the swing phase. On the other hand, it also shows that the path made by the outer (right) foot when in the stance phase is somewhat shorter than the path made by the inner (left) foot when in the stance phase. Figure 9 offers an elaborated explanation for intentionally exaggerated turning conditions – we present a situation during stance or swing phases where the treadmill turns for 90° in one step, which would be too excessive to be evaluated experimentally, but can be used to get an insight into the development of kinematic patterns. Due to combined linear and angular movements of the rotating treadmill, as well as the placement of outer foot with respect to inner foot, the point of foot contact and the point of foot-off are for the outer foot further apart in the swing phase than in the stance phase. For the inner foot the situation is just the opposite: the point of foot contact and the point of foot off are for the inner foot further apart in the stance phase than in the swing phase.

### Limitations of the study and recommendations for future research

Although the results show high degrees of similarity when comparing pelvis and torso rotation patterns during over ground turning and walking on a rotating treadmill, and further extend the assumption about longer stride length of the outer leg to gait on the rotating treadmill, the results should be further supported with testing with larger population sizes. Additionally, in this study only neurologically intact adults were invited. To reach practical applicability of the rotating treadmill as a device for training turning during walking, we would also need to investigate the effect of such training on walking and control mechanisms in a series of clinical evaluation experiments.

In this exploratory study we focused on the kinematics of the pelvis and torso in the transversal plane as the most representative features of turning during walking. To further investigate the hypothesis that walking on rotating treadmill elicits similar walking mechanisms as over ground turning future work should also investigate the lower limb kinematics when walking on the rotating treadmill and several other spatial (step width, position of center of mass relative to selected anatomical landmarks) and temporal (inner leg support time/outer leg support time, duration of stance phase/duration of swing phase, stride time) parameters.

## Conclusions

In this study we have showed that pelvis rotation and torso rotation are similar when turning over ground when compared to walking on a rotating treadmill. We have also demonstrated that in both modes of turning, the stride length of the outer leg is longer than the stride length of the inner leg. This suggests that walking on a rotating treadmill has the potential to establish training conditions that require similar walking and control mechanisms as those that are typically present in over ground turning.

## Authors’ original submitted files for images

Below are the links to the authors’ original submitted files for images. Authors’ original file for figure 1 Authors’ original file for figure 2 Authors’ original file for figure 3 Authors’ original file for figure 4 Authors’ original file for figure 5 Authors’ original file for figure 6 Authors’ original file for figure 7 Authors’ original file for figure 8 Authors’ original file for figure 9 Authors’ original file for figure 10 Authors’ original file for figure 11
