# Supplementary figures and images for: Kinematics of turning during walking over ground and on a rotating treadmill
Source: J Neuroeng Rehabil. 2014 Aug 23;11:127. doi: 10.1186/1743-0003-11-127 (PMC4155102; doi:10.1186/1743-0003-11-127)

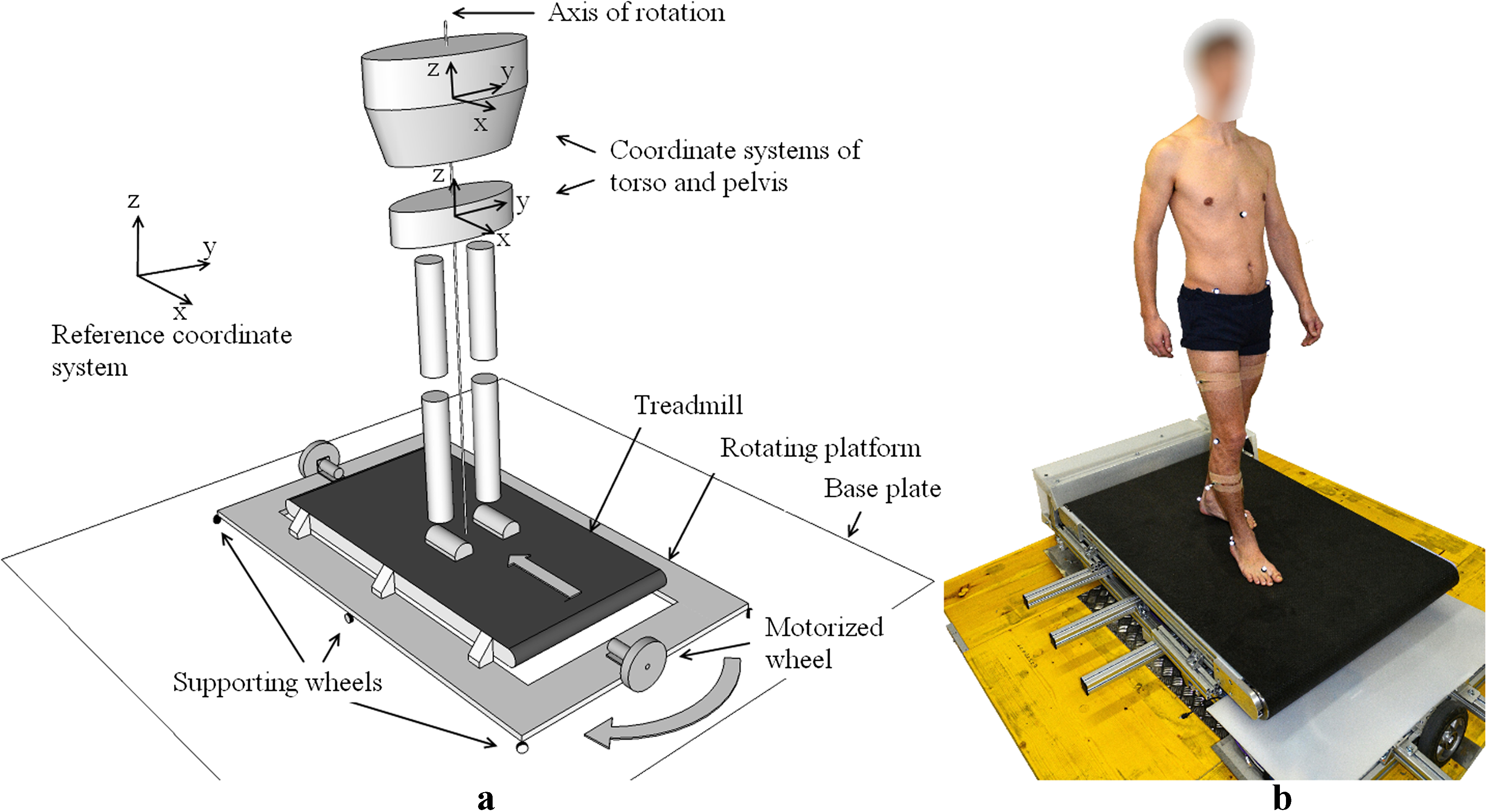

Supplement: Supplementary file 1 — Authors’ original file for figure 1 [file 12984_2013_648_MOESM1_ESM.tiff]

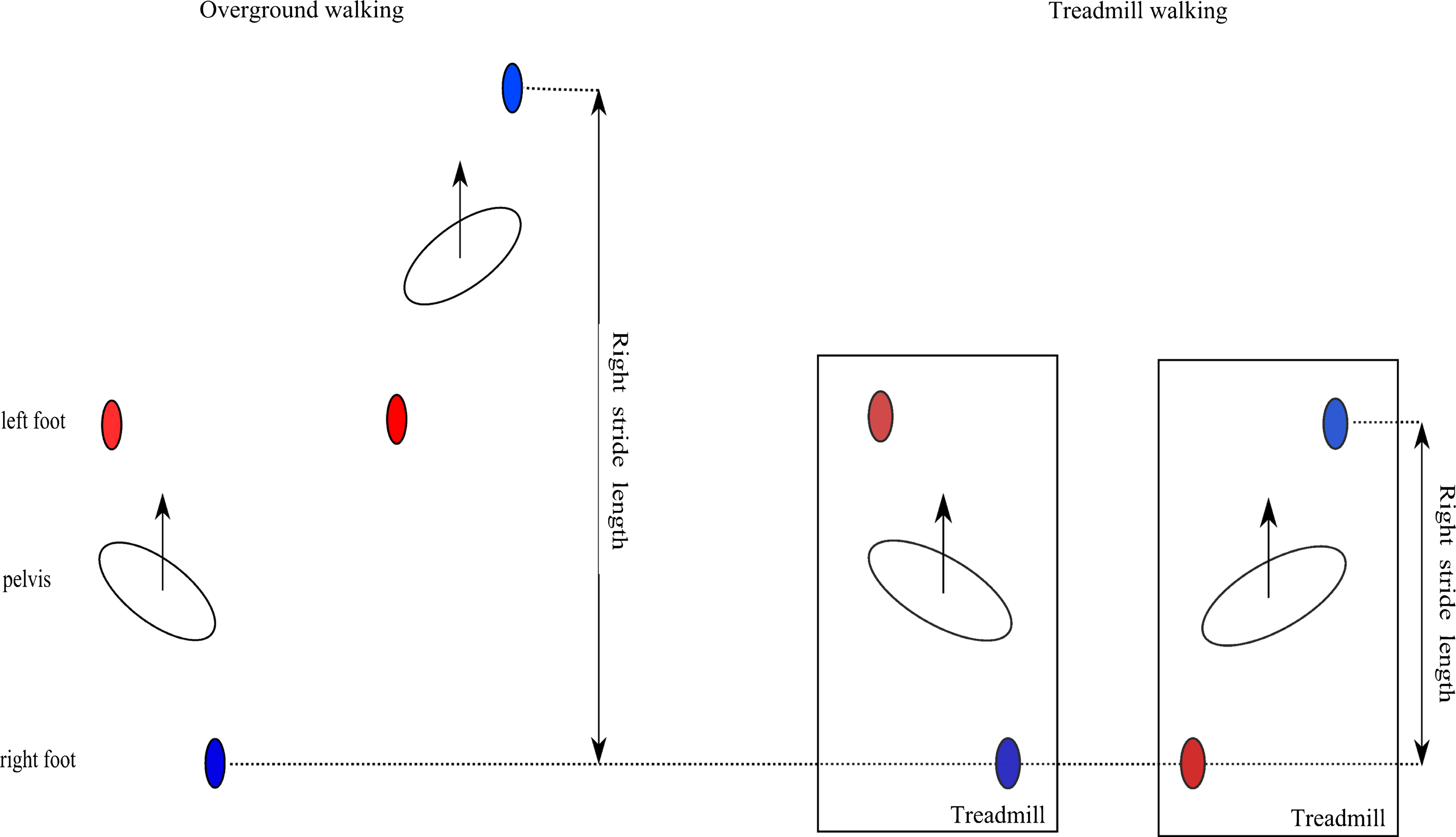

Supplement: Supplementary file 2 — Authors’ original file for figure 2 [file 12984_2013_648_MOESM2_ESM.tiff]

$$ec = \omega_2 v_2$$

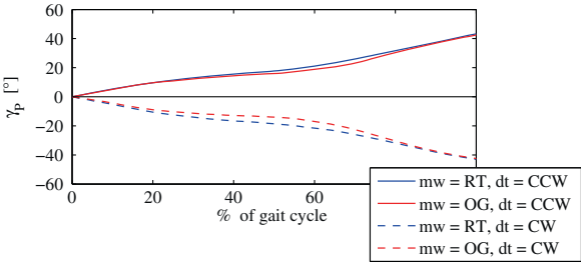

Supplement: Supplementary file 3 — Authors’ original file for figure 3 [file 12984_2013_648_MOESM3_ESM.pdf]

$$ec = \omega_2 v_2$$

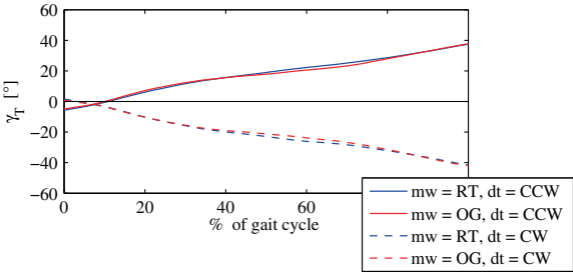

Supplement: Supplementary file 4 — Authors’ original file for figure 4 [file 12984_2013_648_MOESM4_ESM.pdf]

$ec = \omega_1 v_1$ ,  $dt = CCW$

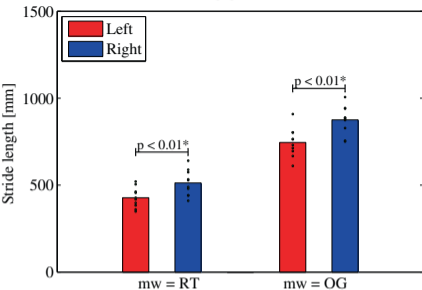

$ec = \omega_1 v_2$ ,  $dt = CCW$

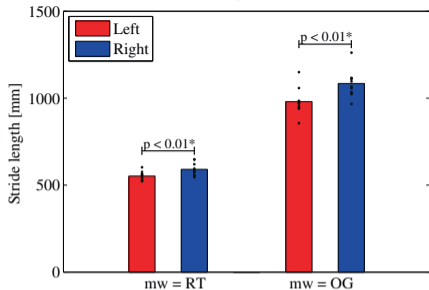

$ec = \omega_2 v_1$ ,  $dt = CCW$

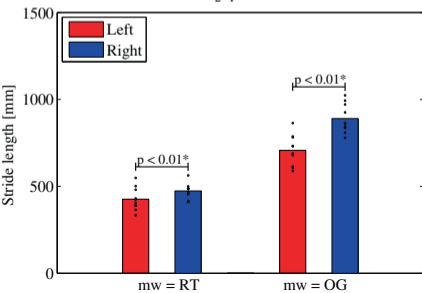

$ec = \omega_2 v_2$ ,  $dt = CCW$

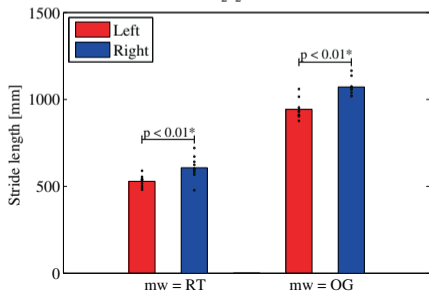

Supplement: Supplementary file 5 — Authors’ original file for figure 5 [file 12984_2013_648_MOESM5_ESM.pdf]

$\omega_1 v_1$ , dt = CW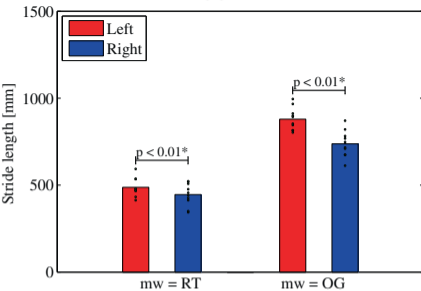 $\omega_1 v_2$ , dt = CW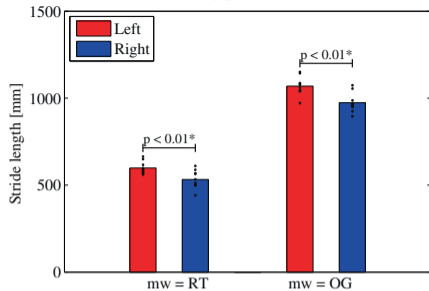 $\omega_2 v_1$ , dt = CW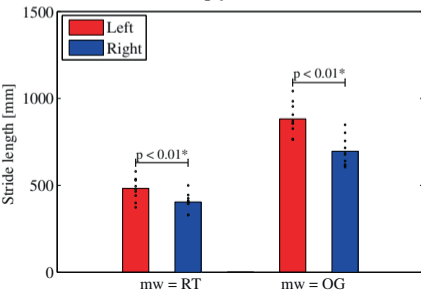 $\omega_2 v_2$ , dt = CW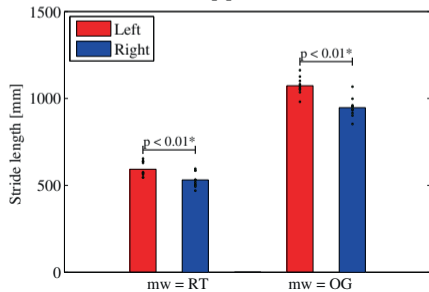

Supplement: Supplementary file 6 — Authors’ original file for figure 6 [file 12984_2013_648_MOESM6_ESM.pdf]

## Overground walking

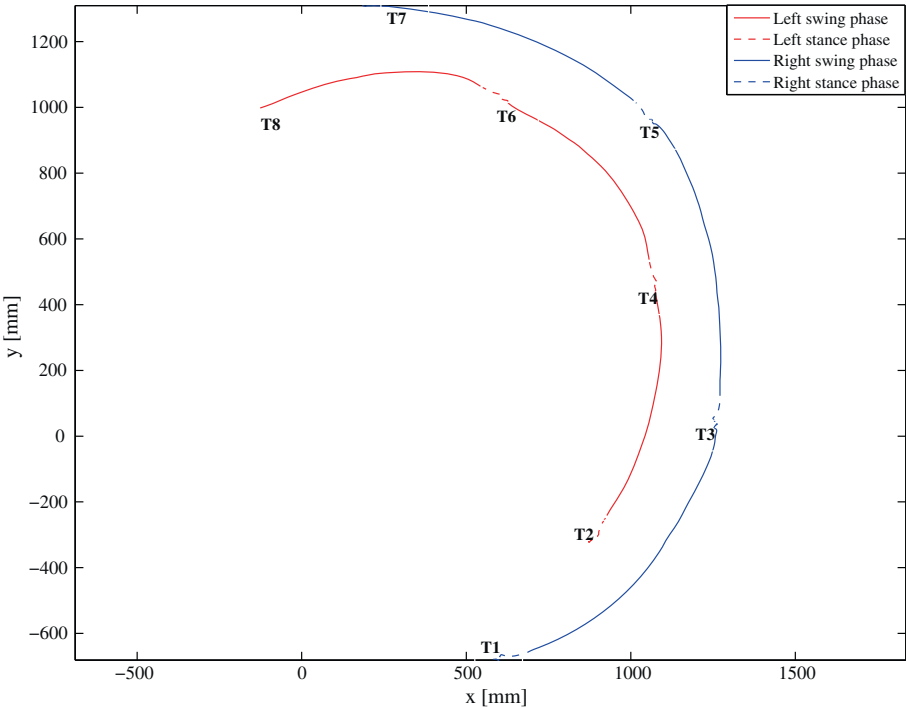

Supplement: Supplementary file 7 — Authors’ original file for figure 7 [file 12984_2013_648_MOESM7_ESM.pdf]

# Treadmill walking

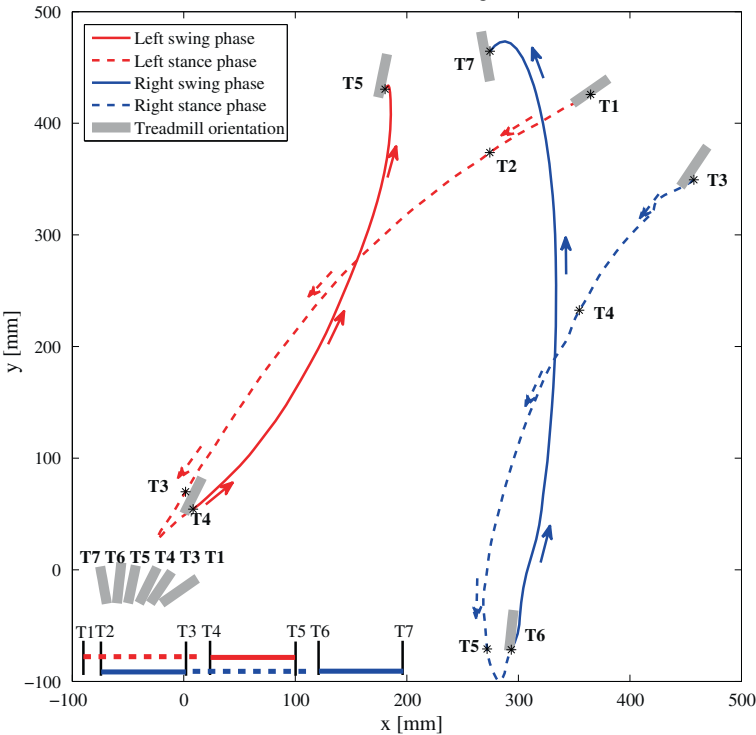

Supplement: Supplementary file 8 — Authors’ original file for figure 8 [file 12984_2013_648_MOESM8_ESM.pdf]

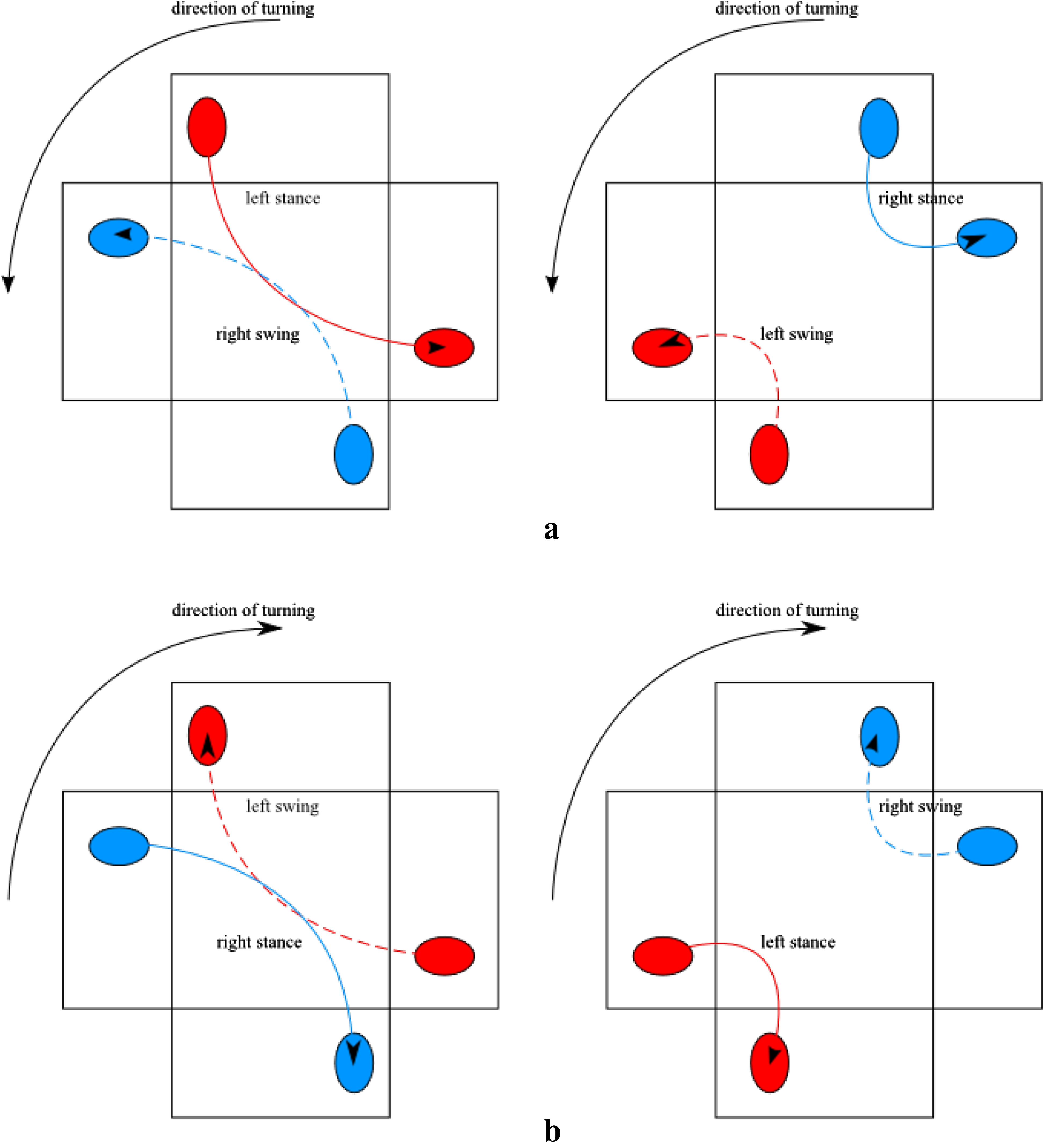

Supplement: Supplementary file 9 — Authors’ original file for figure 9 [file 12984_2013_648_MOESM9_ESM.tif]
